# Maternal Investment Influences Expression of Resource Polymorphism in Amphibians: Implications for the Evolution of Novel Resource-Use Phenotypes

**DOI:** 10.1371/journal.pone.0009117

**Published:** 2010-02-09

**Authors:** Ryan A. Martin, David W. Pfennig

**Affiliations:** Department of Biology, University of North Carolina, Chapel Hill, North Carolina, United States of America; University of Liverpool, United Kingdom

## Abstract

Maternal effects—where an individual's phenotype is influenced by the phenotype or environment of its mother—are taxonomically and ecologically widespread. Yet, their role in the origin of novel, complex traits remains unclear. Here we investigate the role of maternal effects in influencing the induction of a novel resource-use phenotype. Spadefoot toad tadpoles, *Spea multiplicata*, often deviate from their normal development and produce a morphologically distinctive carnivore-morph phenotype, which specializes on anostracan fairy shrimp. We evaluated whether maternal investment influences expression of this novel phenotype. We found that larger females invested in larger eggs, which, in turn, produced larger tadpoles. Such larger tadpoles are better able to capture the shrimp that induce carnivores. By influencing the expression of novel resource-use phenotypes, maternal effects may play a largely underappreciated role in the origins of novelty.

## Introduction

A central goal of evolutionary biology is to understand how novel, complex traits arise [1], [2]. An organism's external environment often plays a critical role in directing both the development and evolution of novel traits. Specifically, the environment may promote the development of novel traits through phenotypic plasticity (see recent review in [3]), and it may promote the evolution of novel traits through genetic accommodation (sensu [1]). Thus, clarifying how the environment influences the expression and evolution of complex traits is crucial for understanding how new traits arise.

Here, we explore the role of the maternal environment in influencing the expression of a novel resource-use phenotype. Understanding how resource-use phenotypes originate is important, because their evolution may permit populations to invade and persist in novel or changing environments [4], [5]. Moreover, the evolution of resource polymorphism – in which alternative morphs showing differential resource use occur in the same population [4] – may represent a critical, early phase in the formation of a new species [1], [4], [6], [7].

We specifically consider how the phenotype *of an individual's mother*, independent of the effects of her genes, influence which resource-use morph the individual will ultimately express. Little is known about whether and how such maternal effects (sensu [8]) mediate resource polymorphism [4], (but see [9], [10] for possible examples). Yet, maternal effects may play a key role in the development and evolution of resource polymorphism, especially in species, such as amphibians, where egg provisioning often constitutes the only maternal investment [11], [12], and where a mother's environment can influence the amount (and, possibly, quality) of provision she allocates to each egg [13]–[16]. Such differential investment can, in turn, profoundly affect her offspring's phenotype (reviewed in [12]). For example, larger females often produce larger eggs [14]–[17], which develop into larger, faster developing tadpoles [15], [18]–[20]. Furthermore, differential investment may mediate plasticity in the expression of offspring traits [9], [19], [21], [22]. In particular, in species where individuals facultatively express alternative resource-use morphs depending upon their environmental circumstances (e.g., see [23]–[25]), offspring that receive greater maternal investment may induce a different resource-use morph than offspring that receive less maternal investment (e.g., see [9], [26]). Thus, maternal effects may often be critical in the expression of resource polymorphism.

Previous research by Pfennig and Martin [10] suggests that a condition-dependent maternal effect mediates differences in the expression of resource polymorphism in different populations of an amphibian. In the present study, we examine the proximate mechanisms underlying such a maternal effect. We also speculate on the role maternal effects may play in the evolution of novel resource-use phenotypes. Before outlining our specific objectives, we first describe our study system in more detail.

### Study System

Mexican spadefoot toads (*Spea multiplicata*: Family Pelobatidae) occur in Mexico and the southwestern U.S. [27]. Their tadpoles are unusual in that they exhibit considerable variation in resource use, morphology, and life history, even within a single clutch. In particular, these tadpoles may develop into either a small, slowly developing tadpole with normal sized jaw muscles used for feeding on detritus at the pond bottom (the “omnivore” morph), or a larger, more rapidly developing tadpole with greatly enlarged jaw muscles used for feeding on anostracan fairy shrimp in open water (the “carnivore” morph), [24], [28]–[30], (for photos of both morphs, see [31]). The carnivore morph is a novel phenotype within the family Pelobatidae that has arisen only in the genus *Spea* [5].

Morph determination is environmentally induced. Tadpoles are born as omnivores, but they may develop into carnivores if they ingest anostracan fairy shrimp early in life [24], [29], [32]. However, there is considerable variation in carnivore production, and some sibships are more prone than others to produce carnivores, even when tadpoles are reared under common conditions [33], [34]. Moreover, because shrimp are limited in most natural ponds [30], competition among tadpoles for the more nutritious shrimp prey [35] critically affects the probability that any particular tadpole will eat shrimp and thus experience the cue that induces the carnivore morph [30], [36].

This resource polymorphism appears to be maintained evolutionarily as an adaptive response to variation among natural ponds in longevity and resource availability. Carnivores are favored in highly ephemeral ponds where shrimp are most abundant and where a carnivore's rapid growth and development increase their likelihood of metamorphosing before their pond dries [24], [30]. Omnivores, by contrast, are favored in longer-lasting ponds, where shrimp tend to be scarce [24]. However, both morphs are often present in the same pond [24], [28], [29]. In such situations, individuals compete for food most with other tadpoles that express the same morphotype [30], [37]. As a result, negative frequency-dependent selection favors the rarer morph [30]. Thus, selection to minimize competition for food can maintain both morphs in the same pond.

An additional selective agent influencing the evolution of resource polymorphism in this system is interspecific competition for food. In southeastern Arizona and southwestern New Mexico, USA, *S. multiplicata* co-occurs with a congener, *S. bombifrons* [31], [38], whose tadpoles outcompete *S. multiplicata* for shrimp and thereby produce a competitively superior carnivore [31]. Selection to lessen competition between these species has led to divergence in resource-use traits, both between species [39] and between sympatric and allopatric populations within each species [31], [38]. In particular, where each species occurs alone, they produce similar, intermediate frequencies of both morphs [40]. However, where they co-occur, *S. multiplicata* produces mostly omnivores, whereas *S. bombifrons* produces mostly carnivores [31], [38], [40]. Experiments reveal that this divergence in morph production reflects selection to lessen interspecific competition for food; i.e., it reflects ecological character displacement [38], [39]. Moreover, differences in morph production between sympatric and allopatric population of *S. multiplicata* persist even when tadpoles are produced and reared under common conditions, suggesting that they are developmentally canalized [31], [38].

Population differences in morph production have also resulted in shifts in adult size [41] and condition [10]. Partly because they produce only the smaller omnivore morph in the presence of *S. bombifrons*, *S. multiplicata* mature as smaller adults in sympatry relative to allopatry [41]. In addition, females from sympatry produce smaller eggs than females from allopatry [10]. Because differences in morph production are maternally inherited and disappear once females have been equilibrated in condition [10], morph production may in part be a result of a condition dependent maternal effect. This maternal effect may be driven by differences in adult body size and maternal investment between allopatric and sympatric *S. multiplicata* [10]. However, the precise mechanism by which a mother's body size and condition influence the resource-use morph of her offspring was (before the present study) unknown.

### Specific Hypotheses and Predictions

The overall goal of our study was to determine the proximate mechanisms underlying a maternal effect influencing the expression of resource polymorphism in *S. multiplicata*. In particular we hypothesized that, through its effects on egg size and ultimately tadpole size, a female's overall body size influences the likelihood that her tadpoles will develop into the novel carnivore morph phenotype.

In order to achieve this goal, we asked whether maternal size predicts the time it takes the offspring to capture and consume a standard amount of shrimp. A tadpole's time to eat shrimp is highly repeatable for individual tadpoles (Spearman correlation between the separate times to eat two consecutive shrimp for 75 *S. multiplicata* tadpoles  = 0.63, *P*<0.0001). More importantly, the amount of time that a tadpole takes to eat a standard quantity of shrimp reliably predicts its propensity to later develop into a carnivore morph: tadpoles that eat shrimp the fastest are ultimately the most likely to express the distinctive large-headed carnivore phenotype [39] (Spearman correlation between the time to eat three shrimp and the degree to which the carnivore morphology is expressed for 130 *S. multiplicata* tadpoles  = −0.36, *P*<0.001). Finding that maternal, but not paternal, size predicts offspring shrimp foraging ability would further suggest that a maternal effect influences offspring shrimp foraging ability, and, by extension, their propensity to produce carnivores. Therefore, we also asked whether paternal size predicts their offspring's time to capture and consume shrimp. In addition, because we hypothesized that females may affect their offspring's morph determination by influencing offspring size, we asked if tadpole size also predicts shrimp foraging ability.

Next, because we found that the largest females produced the most carnivore-like offspring and larger tadpoles were more carnivore-like than smaller tadpoles (see Results), we sought to determine if this result reflected differences among females in maternal investment. We specifically sought to determine if larger females made a greater investment in egg size than smaller females, and if larger eggs produced correspondingly larger tadpoles. Larger tadpoles may be more likely to develop into carnivores, in part, because their greater size gives them an advantage when competing with smaller tadpoles for the shrimp that induce carnivore-morph expression [36].

## Materials and Methods

*Spea multiplicata* bred for these experiments were collected from four breeding aggregations (and from the road nearby) near Rodeo, New Mexico. All animals were transported to the University of North Carolina at Chapel Hill, where they were housed and maintained on identical diets for at least six months before use in the experiments below. The variation in female body size (SVL) used in the experiments described below (49.73±3.23, n = 24) was similar to the variation in female size found in nature (44.30±3.67, n = 260; Pfennig and Pfennig 2005).

This study was conducted in compliance with the Public Health Service (PHS) policy on Humane Care and Use of Laboratory Animals, the Amended Animal Welfare Act of 1985, and the regulations of the United States Department of Agriculture (USDA) under the supervision of the Institutional Animal Care and Use Committee (IACUC) at the University of North Carolina at Chapel Hill under application #06-047.0-A. Field collections were conducted under New Mexico collecting permit 1857.

### Relationship between Maternal Size, Paternal Size, Offspring Size and Offspring Foraging Behavior

We generated tadpoles to examine the relationships between maternal size, paternal size, offspring size and offspring foraging behavior by breeding 20 pairs of *S. multiplicata*, which were randomly paired with their mate with respect to body size (SVL). We chose females with fully developed eggs for breeding, by visually inspecting each female's clutch through her skin. Immediately before breeding, we measured each toad's snout-vent length (SVL) and injected each with 0.07 ml of 0.1 mM gonadotropin releasing hormone to induce breeding. We then placed each male-female pair in an 11.3 L tank filled with dechlorinated water and left them undisturbed overnight. The next day we removed the adult toads and left the fertilized eggs within the tanks where they were oviposited. *Spea* tadpoles hatch approximately 24 hours after oviposition and fertilization and are free swimming approximately 12 hours after hatching. Five days after the breeding, we thinned out the tadpoles to approximately 50 tadpoles per 11.3 L plastic tank, keeping tadpoles from the same clutch together, and fed them crushed fish food *ad libitum* (Wardley cichlid floating pellets).

Twelve days after the breeding, we measured the ability of 21 tadpoles from each of 10 randomly chosen families to capture and consume shrimp (two tadpoles died prior to the end of the experiment and were therefore not included in the subsequent analysis). We used tadpoles from only 10 families to maximize the number of tadpoles we could test from each family. We placed each tadpole separately in an individually numbered round, opaque, plastic container (12 cm in diameter and 6 cm deep) filled with 600 mL of de-chlorinated water (kept at 23°C). We arranged the cups on a table randomly with respect to family. We allowed the tadpoles to acclimate to their new surroundings for twenty-four hours, during which time they were fed crushed fish food (Wardley cichlid floating pellets) to ensure that all tadpoles were equally satiated. The next day, we placed into each container three, live brine shrimp (*Artemia* sp., 10 mm total length; brine shrimp are similar to the fairy shrimp on which *Spea* tadpoles prey in natural ponds and can induce carnivore-like morphology; [39]. We then observed the tadpoles continuously and recorded the time each took to capture and consume all three shrimp. To determine if tadpole size, maternal size, and paternal size predicted offspring shrimp foraging ability we fit a linear mixed effect model to the data with maternal size (mother's SVL), paternal size (father's SVL) and tadpole size (SVL) as fixed effects. The response variable, the time for each tadpole to capture and consume three shrimp, was natural log transformed to meet the assumptions of normality. In addition, we included the random effect of family identity, to account for non-independence among tadpoles from the same family, nested within the random effect of population, using restricted maximum likelihood (REML). Statistical analyses were performed in the nlme package in R version 2.8.1.

### Relationship between Maternal Size and Egg Size

To examine the relationship between maternal size and egg size we collected 10–25 eggs, from each of the 20 clutches generated in the breeding described above, within a few hours of oviposition. We immediately preserved these eggs in 10% buffered formalin. At a later date we used a Leica MZ16 dissecting microscope with a Leica DFC480 digital camera to photograph the eggs at 10x magnification. We determined the Gosner developmental stage of each egg [42] and measured its diameter by using ImageJ 1.37v [43]. We assumed that each egg was spherical and calculated its volume from the formula 4/3πr^3^, where r is the radius. We only included eggs in the same Gosner stage in our analysis (stage 12, [42]) because egg volumes change during development and are not directly comparable across developmental stages [16], [44], [45]. In addition, it is important to note that preservation in formalin can affect egg size [45]. Therefore, we likely did not measure the true egg size of our formalin preserved eggs. However, because we were interested in the relationship between relative egg size and maternal size, changes in egg size caused by fixation in formalin were unlikely to qualitatively affect our results.

Because we specifically predicted that larger females would produce larger eggs we employed a one-tailed test. Specifically, we fit a linear mixed effect model to the data to determine if maternal size (SVL) predicted egg volume. We treated maternal size as a fixed effect. In addition, we included the random effect of family identity, to account for non-independence among eggs from the same clutch, nested within the random effect of population, using restricted maximum likelihood (REML). Statistical analyses were performed in the nlme package in R version 2.8.1.

### Relationship between Egg Size and Tadpole Size

To examine the effect of egg size on tadpole size (where again, egg size served as a measure of maternal investment), we measured the size of individual eggs from the single cell stage, and again as tadpoles seven days after oviposition. We chose to re-measure the tadpoles seven days after oviposition because carnivores can first be found in natural ponds around this time [46]. We generated four clutches from four pairs of *S. multiplicata*, from separate populations, each on a separate day, using procedures described above. We collected eggs within twenty minutes of their laying, and before they developed into the two-cell stage (Gosner stage 3, [42]). We continued to collect eggs periodically as new eggs were laid. We de-jellied eggs with a 2% L-cysteine solution for no longer than 2 minutes and washed them three times with an isotonic buffer [0.1x Marc's Modified Ringers (MMR) pH 7.5 (100 mM NaCl, 2 mM CaCl_2_, 1 mM MgCl_2_, 5 mM HEPES, 2 mM KCl)]. We then placed each egg in a petri dish filled with 40 milliliters of an isotonic buffer (0.1x MMR) and measured each egg at the one cell stage as described above. In addition, we measured each tadpole's body size (SVL) with handheld digital calipers seven days after oviposition (number of individuals measured per clutch  = 26±14). Between measurements, eggs and tadpoles were kept in an environmental chamber at 25° C on a 14L: 10D cycle. Eggs were reared in 0.1x MMR and transferred, when free swimming, to dechlorinated tap water that was changed daily. Tadpoles were fed crushed fish food (Wardley cichlid floating pellets) and brine shrimp *ad libitum*, starting 36 hours after oviposition.

We calculated egg volume, tadpole size and developmental stage as described above. Because we specifically predicted that larger eggs would produce larger tadpoles, we employed a one-tailed test. Specifically, we fit a linear mixed effect model to the data. Initial egg size was treated as a fixed effect, and tadpole size (SVL) at seven days post-laying was the response measure. We included family identity as a random effect, using restricted maximum likelihood (REML), to control for the possibility that differences among families drove the relationship between egg size and tadpole size. Statistical analyses were performed in the nlme package in R version 2.8.1.

## Results

### Relationship between Maternal Size, Paternal Size, Offspring Size and Offspring Foraging Behavior

**Figure 1:**
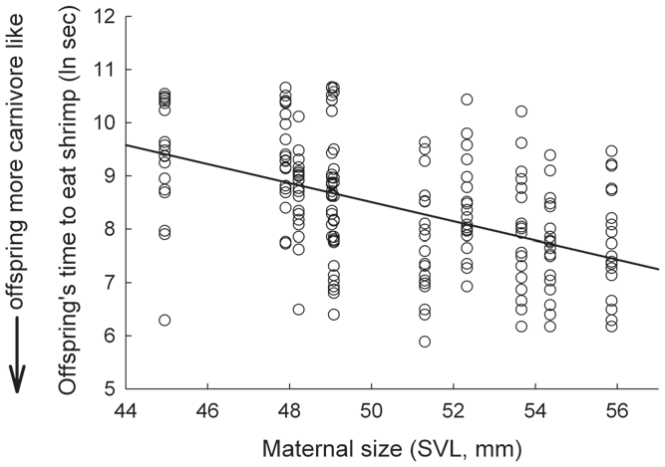
The relationship between maternal size and offspring foraging ability. Shown is maternal size (SVL) and their offspring's time to eat three shrimp (ln sec) for 10 families of *S. multiplicata*. Shrimp foraging ability is a proxy for carnivore morph production, where faster shrimp eating times suggest a higher propensity for a tadpole to develop as a carnivore. Data points for individual offspring are shown.

**Figure 2:**
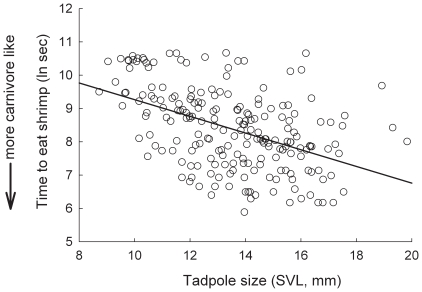
The relationship between tadpole size and foraging ability. Shown is tadpole size (SVL) and the time taken to eat three shrimp (ln sec) for 208 *S. multiplicata* tadpoles from 10 families. Shrimp foraging ability is a proxy for carnivore morph production, where faster shrimp eating times suggest a higher propensity for a tadpole to develop as a carnivore.

Both maternal size (SVL, (*F*_1,4_  = 18.079, *P* = 0.013)) and tadpole size (SVL, *F*_1,197_  = 55.845, *P*<0.0001) significantly predicted offspring foraging behavior. Indeed, there was a significant, negative relationship between the time it took to capture and consume a standard quantity of shrimp and both maternal size (Fig. 1; slope  = −0.14 ± s.e. 0.048) and tadpole size (Fig. 2; slope  = −0.217 ± s.e. 0.031). By contrast, there was no significant relationship between paternal size (SVL) and offspring foraging behavior (*F*_1,4_  = 1.428, *P* = 0.298). In addition, the random effect of family identity accounted for ∼16% of the remaining variation in offspring foraging behavior. In contrast, the random effect of population accounted for <<1% of the remaining variation in offspring foraging behavior.

### Relationship between Maternal Size and Egg Size

**Figure 3:**
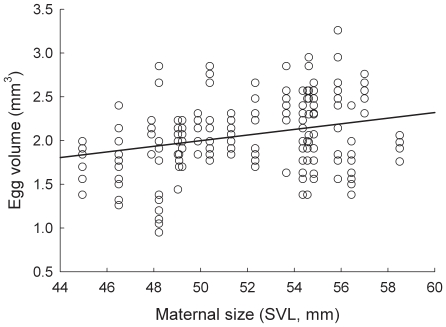
The relationship between maternal size and egg volume. Shown is maternal size (SVL) and egg volume for 20 families of *S. multiplicata*. Data points for individual eggs are shown.

Maternal size (SVL) significantly predicted egg volume (*F*_1,14_  = 6.22, *P* = 0.013 for a one-tailed test). In particular, there was a significant, positive relationship between maternal size (SVL) and egg volume (Fig. 3; slope  = 0.022 ± s.e. 0.09). Thus, larger females produced larger eggs. In addition, the random effect of family identity accounted for ∼31% of the remaining variation in egg size. In contrast the random effect of population accounted for <<1% of the remaining variation in offspring foraging behavior.

### Relationship between Egg Size and Tadpole Size

**Figure 4:**
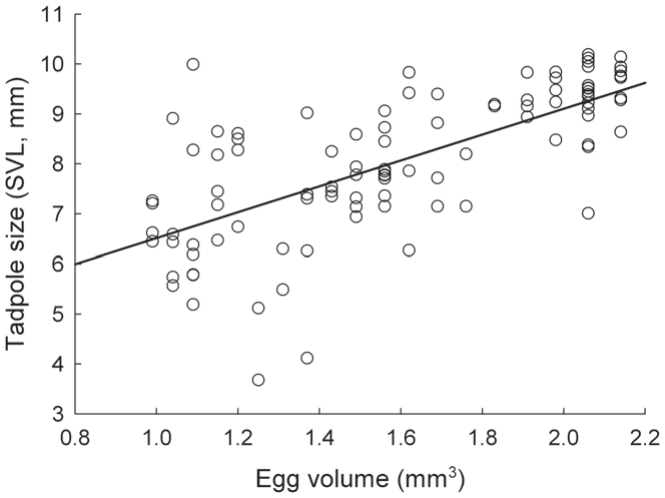
The relationship between egg volume and tadpole body size. Shown is egg volume and tadpole body size (SVL) measured at seven days after oviposition, for 102 *S. multiplicata* individuals from four families.

There was a significant relationship between egg size and tadpole size seven days after oviposition (*F*_1, 97_  = 3.175, *P* = 0.039 for a one tailed test). In particular, there was a significant, positive relationship between egg volume and the size (SVL) of tadpoles emerging from each egg (Fig. 4; slope  = 0.829 ± s.e. 0.425). Thus, larger eggs produced larger tadpoles. In addition, the random effect of family identity accounted for 36% of the remaining variation in tadpole size.

## Discussion

We evaluated the proximate mechanisms by which a maternal effect influences the expression of a novel resource-use phenotype, and thereby, resource polymorphism. Specifically, we asked how a female spadefoot toad's body size influences the propensity of her tadpoles to develop into a distinctive carnivore morph, as opposed to the normal omnivore morph. Our results suggest that the expression of the carnivore morph is indeed influenced by maternal phenotype. Specifically, female size was significantly negatively correlated with the time it took offspring to capture and consume shrimp (Fig. 1), which, in turn, is significantly negatively correlated with an individual's propensity to become a carnivore [39]. Thus, large females produced large offspring with a greater propensity to become carnivores (Fig. 2). In contrast, there was no effect of paternal size on offspring foraging behavior.

It might be contended that a purely genetic correlation between female size and tadpole size, rather than a maternal effect, explains why larger females produce offspring with a greater propensity to become carnivores. However, such a genetic correlation is unlikely the main factor explaining variation in shrimp foraging behavior, because the relationship between female size and offspring morph determination appears to be condition dependent: altering a female's size (through differential feeding in the lab) affects the propensity of her offspring to develop as carnivores [10]. Both short and long term changes in female size and condition are known to affect maternal investment and tadpole phenotype in anurans [13]–[15], [18]. Indeed, the tendency for large mothers to produce tadpoles with a greater propensity to become carnivores appears to reflect differences in maternal investment. Larger females invested in larger eggs (Fig. 3), which produced correspondingly larger tadpoles (Fig. 4). Larger tadpoles, in turn, are able to handle shrimp more efficiently (Fig. 2) and may be able to consume them faster and earlier in development than smaller tadpoles. Larger tadpoles may thereby acquire more of the cue – shrimp ingestion – that induces the carnivore morph [24], [29], [32]. In natural ponds, larger tadpoles may be especially likely to develop into carnivores by outcompeting smaller tadpoles for shrimp [36], which is a limiting resource [40].

The lack of a significant relationship between offspring foraging behavior and paternal size does not rule out environmental or genetic parental effects uncorrelated with parental size. Indeed, the amount of variation accounted for by family identity in offspring foraging behavior, egg size, and tadpole size (see Results), suggests that additional parental effects contribute to these traits and to the expression of resource polymorphism. Further studies are needed to explore the possible contributions (if any) of environmental or genetic parental effects uncorrelated with parental size.

Because our results are correlative, we did not directly test the relationship between maternal phenotype, maternal investment, and offspring morph determination. Therefore, it would be valuable to experimentally test the hypothesis suggested by our study; that maternal size influences offspring morph determination via maternal investment. This hypothesis could be tested experimentally by 1) manipulating the degree of maternal investment to individual eggs through yolk removal [e.g., 47] and 2) through nuclear transplantation [48], where the original nuclear genetic material of an egg is destroyed and replaced with the nuclear genetic material from a donor cell. These two approaches could disentangle the affects of maternal investment on the expression of resource polymorphism from other environmental and genetic parental effects.

Nevertheless, our data suggest that maternal effects may play an important role in mediating resource polymorphism. Although our study focused on how maternal effects influence resource polymorphism through differential maternal investment, maternal effects mediated by oviposition decisions, such as where eggs are laid, may also be important. For example, the expression of resource polymorphism can be influenced by numerous environmental cues, such as conspecific density [23], [49], [50], degree of relatedness among conspecifics [51], [33], and the type and quality of prey [24], [25], [52], [53]. How offspring experience each of these factors may depend, in turn, on where a female deposits her eggs. Thus, maternal effects that are manifested as differential oviposition decisions could also influence the expression of resource polymorphism.

Although maternal effects are increasingly viewed as being important in driving rapid phenotypic change within populations (reviewed in [54], [55]), they may also promote rapid divergence *between* populations that differ consistently in exposure to the environmental stimuli that influence the maternal effect [54], [55], (e.g., see [56], [57]). Indeed, the relationship between maternal size and investment may reinforce character displacement and population divergence in spadefoot toads. Partly because they produce only the smaller omnivore morph in the presence of *S. bombifrons*, *S. multiplicata* mature as smaller adults in sympatry relative to allopatry (see Study System; [41]). However, *S. multiplicata* females from sympatry that developed as omnivores when they were young would grow up not only smaller, but – because of the maternal effect – they would also produce mostly omnivores in the next generation (smaller females produce more omnivores; Fig. 1, [10]). This relationship between female size and offspring morph determination – in which smaller females produce mostly omnivores, which likely mature as smaller females that produce mostly omnivores in the next generation – could produce a self-reinforcing, epigenetic cycle that accelerates the evolution of character displacement and population divergence (see [10] for a more detailed discussion).

In addition to mediating ecological divergence between populations and species, maternal effects may also promote the origin of complex traits. Long-standing theory suggests that novel traits may often begin as environmentally initiated phenotypic change [1], [58]–[60]. According to this theory, environmentally triggered variants (such as differential trait expression induced by variation in maternal investment) may, by chance, improve an organism's fitness under stressful conditions [58], [59], [61]. If heritable variation exists among individuals in tendency to produce the newly favored trait, then selection should favor those alleles or gene combinations that best stabilize, refine, and extend the new trait's expression (a process known as genetic accommodation; [1]). Over evolutionary time, a trait that was initially triggered by the environment may either become canalized or become part of an alternate phenotype controlled by a developmental switch (e.g., see [62], [63]).

The resource polymorphism found in spadefoot toads may have evolved under such a scenario [5]. Rapid growth and development is critical for escaping the ephemeral ponds in which *Spea* breed [24], [30]. Ancestral *Spea* tadpoles that could occasionally consume fairy shrimp would likely have experienced enhanced growth and development. Because they consumed the more nutritious shrimp resource as tadpoles, females would have reached larger body size as adults, and some may have produced larger eggs, resulting in larger tadpoles, which were, in turn, better able to capture and consume shrimp. Genetic accommodation (see above) could have then refined the existing variation in shrimp foraging ability to favor the evolution and elaboration of the distinctive carnivore phenotype. In this way, a maternal effect could have played a role in the evolution of a novel, complex trait.

Traits whose expression is mediated by a maternal effect may be especially prone to undergo genetic accommodation [1], [64], [65]. Because maternal effects often impinge on many, genetically diverse, offspring, they tend to be tested in numerous genetic backgrounds, thereby increasing the chances of genetic accommodation occurring. Moreover, because they can persist across multiple generations [66], [67], maternal effects provide more frequent, recurrent opportunities for genetic accommodation to occur. For example, novel phenotypes may often evolve as an adaptive response to variation in body size stemming from differential maternal investment and/or oviposition decisions. Indeed, many species have evolved novel specializations to variation in body size, such as carnivore/cannibal and omnivore/typical morphs in various larval amphibians species [23], [25], [29], [68], benthic and limnetic morphs in various fish species (reviewed in [69], [70]), fighting and nonfighting male morphs in certain insect and fish species (reviewed in [71], [72]), and castes in eusocial species (reviewed in [1], [73]). In many cases, these alternative morphs may have started out as environmentally induced size variants that subsequently evolved novel resource-use or reproductive specializations through genetic accommodation. Thus, again, maternal effects may promote the evolution of novel phenotypes.

## References

[1] West-Eberhard MJ (2003). Developmental Plasticity and Evolution..

[2] Moczek AP (2008). On the origin of novelty in development and evolution.. Bioessays.

[3] Gilbert SF, Epel D (2009). Ecological Developmental Biology..

[4] Smith TB, Skúlason S (1996). Evolutionary significance of resource polymorphisms in fishes, amphibians, and birds.. Annual Review of Ecology and Systematics.

[5] Ledón-Rettig C, Pfennig DW, Nascone-Yoder N (2008). Ancestral variation and the potential for genetic accommodation in larval amphibians: Implications for the evolution of novel feeding strategies.. Evolution and Development.

[6] West-Eberhard MJ (1989). Phenotypic plasticity and the origins of diversity.. Annual Review of Ecology and Systematics.

[7] Pfennig DW, McGee M (2009). Resource polyphenism increases species richness: a test of the hypothesis.. Philosophical Transaction of the Royal Society of London, Series B.

[8] Mousseau TA, Fox CW (1998). The adaptive significance of maternal effects.. Trends in Ecology and Evolution.

[9] Michimae H, Nishimura K, Tamori Y, Wakahara M (2009). Maternal effects on phenotypic plasticity in larvae of the salamander *Hynobius retardatus*.. Oecologia.

[10] Pfennig DW, Martin RA (2009). A maternal effect mediates rapid population divergence and character displacement in spadefoot toads.. Evolution.

[11] Bernardo J (1996). The particular maternal effect of propagule size, especially egg size: patterns, models, quality of evidence and interpretations.. The American Zoologist.

[12] Kaplan RH, Mousseau TA, Fox CW (1998). Maternal effects, developmental plasticity, and life history evolution: An amphibian model.. Maternal Effects as Adaptations.

[13] JØrgenson CB (1982). Factors controlling the ovarian cycle in a temperate zone anuran, the toad *Bufo bufo*: food uptake, nutrional state, and gonadotropin.. Journal of Experimental Zoology.

[14] Kaplan RH (1987). Developmental plasticity and maternal effects of reproductive characteristics in the frog, *Bombino orientalis*.. Oecologia.

[15] Kaplan RH (1989). Ovum size plasticity and maternal effects on the early development of the frog, *Bombina orientalis*, in a field population in Korea.. Functional Ecology.

[16] Kaplan RH, King EG (1997). Egg size is a developmentally plastic trait: evidence from long term studies in the frog *Bombina orientalis*.. Herpetologica.

[17] Kaplan RH, Salthe SN (1979). The allometry of reproduction: an empirical view in salamanders.. The American Naturalist.

[18] Kaplan RH (1992). Greater maternal investment can decrease offspring survival in the frog *Bombina orientalis*.. Ecology.

[19] Parichy DM, Kaplan RH (1992). Maternal effects on offspring growth and development depend on environmental quality in the frog *Bombina orientalis*.. Oecologia.

[20] Loman J (2002). Microevolution and maternal effects on tadpole *Rana temporaria* growth and development rate.. Journal of Zoology.

[21] Parichy DM, Kaplan RH (1995). Maternal investment and developmental plasticity: Functional consequences for locomotor performance of hatchling frog larvae.. Functional Ecology.

[22] Kaplan RH, Phillips PC (2006). Ecological and developmental context of natural selection: maternal effects and thermally induced plasticity in the frog *Bombina orientalis*.. Evolution.

[23] Collins JP, Cheek JE (1983). Effect of food and density on development of typical and cannibalistic salamander larvae in *Ambystoma tigrinum nebulosum*.. American Zoologist.

[24] Pfennig DW (1990). The adaptive significance of an environmentally-cued developmental switch in an anuran tadpole.. Oecologia.

[25] Michimae H, Wakahara, M (2002). A tadpole-induced polyphenism in the salamander *Hynobius retardatus*.. Evolution.

[26] Maret TJ, Collins JP (1994). Individual responses to population size structure: the role of size variation in controlling expression of a trophic polyphenism.. Oecologia.

[27] Stebbins RC (2003). A field guide to western reptiles and amphibians: 3d edition..

[28] Bragg AN (1965). Gnomes of the night: the spadefoot toads..

[29] Pomeroy LV (1981). Developmental polymorphism in the tadpoles of the spadefoot toad *Scaphiopus multiplicatus*. PhD dissertation..

[30] Pfennig DW (1992). Polyphenism in spadefoot toads as a locally adjusted evolutionary stable strategy.. Evolution.

[31] Pfennig DW, Murphy PJ (2002). How fluctuating competition and phenotypic plasticity mediate species divergence.. Evolution.

[32] Storz BL (2004). Reassessment of the environmental mechanisms controlling developmental polyphenism in spadefoot toad tadpoles.. Oecologia.

[33] Pfennig DW, Frankino WA (1997). Kin-mediated morphogenesis in facultatively cannibalistic tadpoles.. Evolution.

[34] Pfennig DW (1999). Cannibalistic tadpoles that pose the greatest threat to kin are most likely to discriminate kin.. Proceedings of the Royal Society of London, Series B.

[35] Pfennig DW (2000). Effect of predator-prey phylogenetic similarity on the fitness consequences of predation: a trade-off between nutrition and disease?. The American Naturalist.

[36] Frankino WA, Pfennig DW (2001). Condition-dependent expression of trophic polyphenism: effects of individual size and competitive ability.. Evolutionary Ecology Research.

[37] Martin RA, Pfennig DW (2009). Disruptive selection in natural populations: the roles of ecological specialization and resource competition.. The American Naturalist.

[38] Pfennig DW, Murphy PJ (2000). Character displacement in polyphenic tadpoles.. Evolution.

[39] Pfennig DW, Rice AM, Martin RA (2007). Field and experimental evidence for competitions role in phenotypic divergence.. Evolution.

[40] Pfennig DW, Rice AM, Martin RA (2006). Ecological opportunity and phenotypic plasticity interact to promote character displacement and species coexistence.. Ecology.

[41] Pfennig KS, Pfennig DW (2005). Character displacement as the “best of a bad situation”: fitness trade-offs resulting from selection to minimize resource and mate competition.. Evolution.

[42] Gosner KL (1960). A simplified table for staging anuran embryos and larvae with notes on identification.. Herpetologica.

[43] Rasband WS (1996–2007). ImageJ.. Bethesda, National Institute of Health.

[44] Kaplan RH (1979). Ontogenetic variation in “ovum” size in two species of *Ambystoma*.. Copeia 1979:348-350.

[45] King EG, Kaplan RH (1997). Estimating ovum size in amphibians: egg size increases differently among species during early development.. Journal of Herpetology.

[46] Storz BL, Travis J (2007). Temporally dissociated, trait-specific modifications underlie phenotypic polyphenism in *Spea multiplicata* tadpoles, which suggests modularity.. TSW Development & Embryology.

[47] Sinervo B (1990). The evolution of maternal investment in lizards: and experimental and comparative analysis of egg size and its effects on offspring performance.. Evolution.

[48] Briggs R, King TJ (1952). Transplantation of living nuclei from blastula cells into enucleated frog eggs.. Proceedings of the National Academy of Sciences.

[49] Nishihara A (1996). Effects of density on growth of head size in larvae of the salamander *Hynobius retardatus*.. Copeia 1996:478-483.

[50] Hoffman EA, Pfennig DW (1999). Proximate cause of cannibalistic polyphenism in larval tiger salamanders.. Ecology.

[51] Pfennig DW, Collins JP (1993). Kinship affects morphogenesis in cannibalistic salamanders.. Nature.

[52] Wainwright PC, Osenberg CW, Mittelbach GG (1991). Trophic polymorphism in the pumpkinseed sunfish (*Lepomis gibbosus* Linnaeus): effects of environment on ontogeny.. Functional Ecology.

[53] Loeb MLG, Collins JP, Maret TJ (1994). The role of prey in controlling expression of a trophic polymorphism in *Ambystoma tigrinum nebulosum*.. Functional Ecology.

[54] Rossiter MC (1996). Incidence and consequences of inherited environmental effects.. Annual Review of Ecology and Systematics.

[55] Räsänen K, Kruuk EB (2007). Maternal effects and evolution at ecological time scales.. Functional Ecology.

[56] Badyaev AV, Hill GE, Beck ML, Derven AA, Duckworth RA (2002). Sex biased hatching order and adaptive population divergence in a passerine bird.. Science.

[57] Räsänen K, Laurila A, Merilä J (2003). Geographic variation in acid stress tolerance of the moor frog *Rana arvalis* II. Adaptive maternal effects.. Evolution.

[58] Baldwin JM (1896). A new factor in evolution.. The American Naturalist.

[59] Schmalhausen II (1949). Factors of Evolution..

[60] Waddington CH (1959). Canalization of development and genetic assimilation of acquired characters.. Nature.

[61] Badyaev AV (2005). Stress-induced variation in evolution: from behavioral plasticity to genetic assimilation.. Proceedings of the Royal Society of London, Series B.

[62] Gomez-Mestre I, Buchholz DR (2006). Developmental plasticity mirrors differences among taxa in spadefoot toads linking plasticity and diversity.. Proceedings of the National Academy of Sciences.

[63] Suzuki Y, Nijhout HF (2006). Evolution of a polyphenism by genetic accommodation.. Science.

[64] West-Eberhard MJ (2005). Developmental plasticity and the origin of species differences.. Proceedings of the National Academy of Sciences.

[65] Badyaev AV, Oh KP (2008). Environmental induction and phenotypic retention of adaptive maternal effects.. BMC Evolutionary Biology.

[66] Agrawal AA, Laforsch C, Tollrian R (1999). Transgenerational induction of defenses in animals and plants.. Nature.

[67] Plaistow SJ, Lapley CT, Benton TG (2006). Context-dependent intergenerational effects: the interaction between past and present environments and its effect on population dynamics.. The American Naturalist.

[68] Walls SC, Belanger SS, Blaustein AR (1993). Morphological variation in a larval salamander: dietary induction of plasticity in head shape.. Oecologia.

[69] Robinson BK, Wilson DS (1994). Character release and displacement in fish: a neglected literature.. The American Naturalist.

[70] Skúlason S, Snorrason SS, Jónsson B, Magurran AE, May RM (1999). Sympatric morphs, populations and speciation in freshwater fish with emphasis on arctic charr.. Evolution of Biological Diversity.

[71] Hamilton WD, Blum MS, Blum NA (1979). Wingless and fighting males in fig wasps and other insects.. Sexual Selection and Reproductive Competition in Insects.

[72] Gross MR, Sargent RC (1985). The evolution of male and female parental care in fishes.. American Zoologist.

[73] Linksvayer TA, Wade MJ (2005). The evolutionary origin and elaboration of sociality in the aculeate hymenoptera: maternal effects, sib-social effects, and heterochrony.. Quarterly Review Of Biology.

